# Diagnosis of Pregnancy by Radiography

**Published:** 1918-05

**Authors:** 


**Diagnosis of Pregnancy by Radiography.** Albert Weil, Paris Med., Apr. 21, 1917, quotes Lacquerriere as having shown a plate of a foetus of 4½ months, expressing the opinion that this was the earliest limit. The author showed a plate at the fourth month, the diagnostic value being especially marked as the mother was only 15 years old, had an intact hymen and denied the possibility of pregnancy, which could not be established by auscultatory signs.

